# Metabolic diseases and lifestyle factors affect arthritis incidence in old Europeans - a cross analysis from the SHARE project

**DOI:** 10.1016/j.pmedr.2025.103089

**Published:** 2025-04-28

**Authors:** Fanji Qiu, Jinfeng Li, Liaoyan Gan, Kirsten Legerlotz

**Affiliations:** a Movement Biomechanics, Institute of Sport Sciences, Humboldt-Universität zu Berlin, Unter den Linden 6, 10099 Berlin, Germany; b Department of Kinesiology, Iowa State University, Ames, IA 50011, USA; c Alberta International School of Recreation, Sport and Tourism of Beijing Sport University, Beijing Sport University, 572423 Lingshui, China; d Department of Movement and Training Sciences, Institute of Sport Sciences, University of Wuppertal, Gauss street 20, 42119 Wuppertal, Germany

**Keywords:** Arthritis, Life style, Risk factors, epidemiology, patient reported outcome measures

## Abstract

**Objective:**

In the context of global aging, the burden of metabolic diseases and arthritis is escalating, necessitating a more comprehensive understanding of the associations between these diseases. As modifiable factor the effect of lifestyle on the progression of arthritis also needs to be considered. Thus, this study aimed to identify the associations of the number of metabolic diseases (MDs) and lifestyle factors, with Rheumatoid Arthritis (RA) and Osteoarthritis (OA).

**Methods:**

This is a cross analysis of data from European cohort collected between 2017 and 2021. The demographic information, lifestyle factors, and disease data were used for a prospective analysis to explore the impact of MDs on the prevalence of arthritis within the 4-year study period (*n* = 43,085). In addition, a cross-sectional analysis of 9th wave participants (*n* = 66,208) was conducted to investigate the relationship between lifestyle factors and arthritis. Cox regression and binary logistic regression models were employed to explore the relationships between various factors and arthritis.

**Results:**

About 6.52 % and 12.54 % participants developed RA and OA within the 4-year study period. Individuals with MDs exhibited a higher risk of new-onset arthritis compared to no-MDs participants. OA prevalence was positively associated with higher age, higher BMI, less physical activity (PA) and smoking. RA prevalence was positively associated with higher age, higher BMI and less PA.

**Conclusion:**

There is a causal relationship between the number of MDs and new-onset Rheumatoid Arthritis and Osteoarthritis. Arthritis prevention programs should consider metabolic diseases as well as lifestyle factors in patients at risk.

## Highlights

• The number of Metabolic diseases was associated with new-onset arthritis.
• Metabolic disease and sex difference should also be considered in arthritis prevention.
• The associations between different arthritis and lifestyle factors are different.

## Graphical abstract

**Figure:**
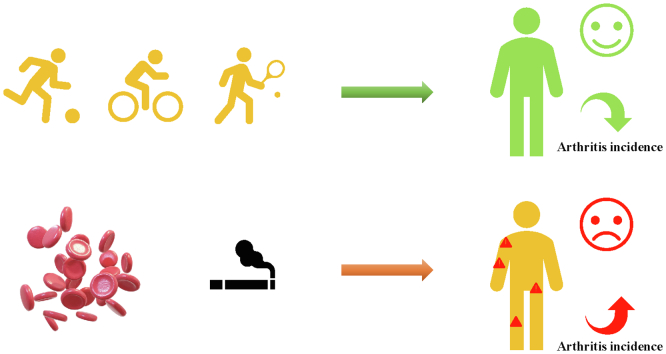

## 1 Introduction

Arthritis is a prevalent chronic disease that leads to functional limitations in patients (Hunter and Bierma-Zeinstra, 2019; Woolf and Pfleger, 2003). Compared to 2020, by the year 2050, the number of Rheumatoid Arthritis (RA) cases worldwide is projected to increase by 80.2 %, while the cases of different types of Osteoarthritis (OA) are expected to grow between 48.6 % to 95.1 % (Collaborators GRA, 2023a; Collaborators GRA, 2023b), making arthritis a significant public health challenge. Arthritis not only imposes a substantial economic burden but is also associated with numerous chronic comorbidities. Metabolic diseases such as hypertension (HTN), diabetes mellitus (DM), and hypercholesterolemia (HC) affect the progression of arthritis (van Breukelen-van der Stoep et al., 2016; Engström et al., 2009), and have been identified as risk factors for both RA and OA (Muhammed et al., 2022; Farnaghi et al., 2017). Consequently, it is anticipated that the number of people affected by arthritis will rise along with the increasing incidence of metabolic diseases (Chew et al., 2023).

In this context, modifiable lifestyle factors need to be considered, as it is known that lifestyle can affect the development of certain diseases including arthritis. Smoking has been identified as a singular risk factor for RA by the data from Global Burden of Disease 2021 across 204 countries and regions, attributing 7.1 % of annual RA incidences (Collaborators GRA, 2023a). Additionally, 32.69 % of RA cases were associated with smoking or low-level alcohol consumption as calculated by a cross-sectional study involving 6437 individuals aged 15 years and older (Ye et al., 2021). An association between alcohol consumption and RA or OA was also observed in a cross-sectional analysis involving 5616 participants with a median age of 66 years, while no association with smoking could be detected in that study (Pengpid and Peltzer, 2023). Moreover, previous alcohol consumption and smoking were associated with higher prevalence in various arthritis types, which was demonstrated by a case-control study including 34,525 adults (Wang et al., 2014). While physical activity has been acknowledged as an effective arthritis management approach (Gwinnutt et al., 2022), several studies were not able to identify an association of PA with arthritis (Pengpid and Peltzer, 2023; Wang et al., 2014). The discrepancies among these findings underline the necessity for further research to fully understand the impact of lifestyle factors on the occurrence of arthritis.

As the population ages, the prevalence of OA and RA is anticipated to rise further (Collaborators GRA, 2023a; Collaborators GRA, 2023b). In 2020, 120 million Europeans were older than 55 years, and of those, approximately 2.88 million Europeans were afflicted with RA, and 3.74 million with OA. By the year 2050, Europeans aged 55 and above are expected to reach around 150 million, and therefore the number of arthritis patients is predicted to increase (Collaborators GRA, 2023a; Collaborators GRA, 2023b; Statistics E., 2020). Thus, understanding the association between the number of metabolic diseases and modifiable lifestyle factors with arthritis particularly in the middle-aged and older populations is crucial for comprehending disease trends and implementing effective disease management strategies.

This study aimed to explore in a four-year prospective study how metabolic diseases and lifestyle factors influence the occurrence of arthritis in European middle-aged and older adults. Our hypotheses were: (Hunter and Bierma-Zeinstra, 2019) the presence of metabolic diseases has an impact on new-onset arthritis, (Woolf and Pfleger, 2003) there exists a dose-response pattern between the number of metabolic diseases and the occurrence of arthritis, varying across different age groups, and (Collaborators GRA, 2023a) level of PA, smoking, drinking are associated with arthritis prevalence.

## 2 Method

### 2.1 Study population and design

**Fig. 1:**
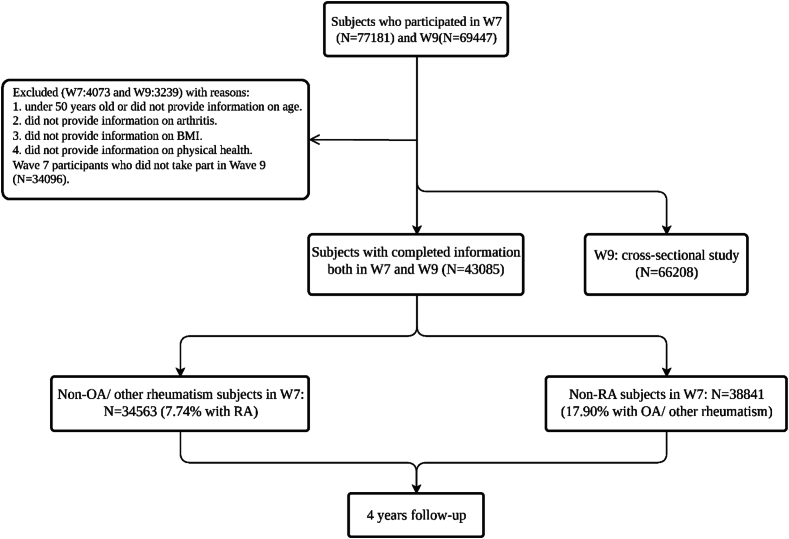
Flow diagram of the study population.

The data used in this study were sourced from the Survey of Health, Aging, and Retirement in Europe (SHARE). SHARE collects longitudinal population data in Europe, to study the impact of multiple factors on the life course (Borsch-Supan et al., 2013). Initiated in 2004, SHARE conducts biennial data collection waves, with nine waves being completed to date. SHARE received ethical approval from the ethics committees of all participating countries, and all subjects provided written informed consent (Börsch-Supan and Jürges, 2005). The Waves 7 to 9 were used for a prospective analysis to explore the impact of MDs on the prevalence of arthritis and included data from individuals aged 50 and above, with participants living in 28 countries (27 European countries and Israel) (Borsch-Supan et al., 2013; SHARE-ERIC, 2024). A cross-sectional analysis of wave 9 data was conducted to investigate the relationship between lifestyle factors and arthritis. Data collection for waves 7 and 9 commenced in 2017 and 2021, respectively, utilizing computer assisted personal interviewing (SHARE-ERIC, 2024; Bergmann et al., 2024). The final dataset of this study included a total of 43,085 community residents aged 50 and older in the longitudinal study and 66,208 in the cross-sectional study (supplemental file). To avoid potential biases caused by missing data, participants with incomplete information were excluded. All participants included in the study provided information regarding arthritis, metabolic diseases, and lifestyle factors. Written informed consent was obtained from all participants (See Fig. 1).

### 2.2 Patient involvement

Given the experimental design of observational studies, it was not appropriate or possible to involve patients or the public in the design, or conduct, or reporting, or dissemination plans of our research.

### 2.3 Demographic information

The participants demographic information included age, sex, and body mass index (BMI).

### 2.4 Information on physical health and lifestyle factors

The physical health-related information that was collected included arthritis (OA/other rheumatism, RA); diabetes mellitus or high blood sugar (DM/HBS); high blood cholesterol (HBC); and high blood pressure or hypertension (HBP/HTN). Information related to lifestyle factors included smoking, alcohol consumption, and physical activity. The collection of participant information was sourced from questionnaires (see supplemental file). The number of metabolic diseases was calculated for each participant.

### 2.5 Statistical analysis

Chi-square tests were utilized to examine the statistical differences in demographic characteristics across different arthritis conditions. Multicollinearity diagnostics was conducted to explore collinearity among variables, with strong collinearity defined as the maximum variance inflation factor (VIF) value over 5 while the tolerance value is less than 0.20 (Rogerson, 2001). Cox regression was employed to investigate the association between the number of metabolic diseases at baseline (wave 7) and new-onset arthritis in wave 9, further stratified into younger (50–65) and older (65+) age groups to explore the relationship between the number of MDs and the occurrence of arthritis in different age cohorts. Time to survival was defined as the interval between two surveys (w7 and w9, 4 years). Multivariable binary logistic regression was used to assess the cross-sectional associations between the dependent variables (RA and OA/other rheumatism) and independent variables (number of MDs), along with covariates (demographic information and lifestyle factors). In the Cox regression model, BMI, Age, and sex (male and female) were included as covariates. In addition to BMI, Age, and Sex, the logistic regression model also considered smoking, alcohol consumption (high, middle, low), and physical activity (vigorous and moderate) as covariates.

Hazard ratios (HR) and odds ratios (OR), along with 95 % confidence intervals and *P* values were calculated. All models were adjusted for covariates. In Cox regression or logistic regression models, the reference category were: participated in vigorous or moderate PA more than once a week, male, no ever daily smoking, no MDs and low alcohol consumption. A *P* value <0.05 was considered statistically significant. The analyses were conducted using SPSS 26.0 (IBM, NY, USA). Based on prevalence rates of 19.32 % for OA and 10.19 % for RA (Qiu et al., 2024), a 95 % confidence interval and a β error of 5 %, the minimum required sample size for this study was calculated to be 240 (Daniel, 1999).

## 3 Results

### 3.1 Participant characteristics

During Wave 7, there were 34,563 participants identified as no-OA/other rheumatism (including 2674 participants with RA) and 38,841 participants identified as no-RA (including 6952 participants with OA/other rheumatism) who also participated in the data collection for Wave 9 (Table 1). About 12,54 % baseline OA-free participants developed OA over 4 years and 6.52 % baseline RA-free participants developed RA over 4 years. Compared to participants without arthritis, participants with new-onset OA/other rheumatism and RA were more likely to be older, female, have a higher BMI, consume less alcohol, and engage in less PA (Table 2). The collinearity diagnostics did not reveal any collinearity among the variables.

**Table 1:** Demographic characteristics of the study participants stated without arthritis in Europe, 2017.

**Variables**	**Non-OA/other rheumatism**	**Non-RA**
**N**	34,563	38,841
**Age [y]**	66.73 ± 8.61	66.92 ± 8.62
**Female [N (%)]**	18,916 (54.7)	21,920 (56.4)
**MDs [N (%)]**		
0	16,170 (46.8)	17,918 (46.1)
1	11,336 (32.8)	12,845 (33.1)
2	5674 (16.4)	6502 (16.7)
3	1383 (4.0)	1576 (4.1)
**BMI [kg/m**^**2**^**]**	27.15 ± 4.52	27.18 ± 4.55

Abbreviation: BMI: body mass index; MDs: metabolic diseases; N: number; OA: osteoarthritis; RA: rheumatoid arthritis; y:year. Age and BMI are presented as mean ± SD.

**Table 2:** Demographic and lifestyle characteristics of the middle-aged and older population by arthritis status in Europe, 2021.

**Variables**	**OA/other rheumatism**				**RA**			
	**All**	**With**	**Without**	***P***	**All**	**With**	**Without**	***P***
**N**	34,563	4334	30,229	–	38,841	2532	36,309	–
**Age [y]**	70.73 ± 8.61	72.76 ± 8.84	70.43 ± 8.54	<0.05	70.92 ± 8.62	73.61 ± 8.82	70.73 ± 8.57	<0.05
**Female [N (%)]**	18,916 (54.7)	2854 (65.9)	16,062 (53.1)	<0.05	21,920 (56.4)	1706 (67.4)	20,214 (55.7)	<0.05
**MDs [N (%)]**								
0	13,540 (39.2)	1305 (30.1)	12,235 (40.5)	<0.05	15,090 (38.9)	636 (25.1)	14,454 (39.8)	<0.05
1	11,800 (34.1)	1598 (36.9)	10,202 (33.7)	<0.05	13,315 (34.3)	925 (36.5)	12,390 (34.1)	<0.05
2	7093 (20.5)	1065 (24.6)	6028 (19.9)	<0.05	8079 (20.8)	729 (28.8)	7350 (20.2)	<0.05
3	2130 (6.2)	366 (8.4)	1764 (5.8)	<0.05	2357 (6.1)	242 (9.6)	2115 (5.8)	<0.05
**BMI [kg/m**^**2**^**]**	27.07 ± 4.65	27.69 ± 5.14	26.98 ± 4.57	<0.001	27.08 ± 4.69	28.09 ± 5.30	27.01 ± 4.63	<0.001
**Smoking [N (%)]**	13,441 (38.9)	1653 (38.1)	11,788 (39.0)	>0.05	15,318 (39.4)	875 (34. 6)	14,443 (39.8)	<0.05
**Alcohol consumption**								
Low	30,539 (88.4)	3936 (90.8)	26,603 (88.0)	<0.05	34,365 (88.5)	2310 (91.2)	32,055 (88.3)	<0.05
Middle	2786 (8.1)	293 (6.8)	2493 (8.3)	<0.05	3140 (8.1)	148 (5.9)	2992 (8.2)	<0.05
High	1238 (3.6)	105 (2.4)	1133 (3.8)	<0.05	1336 (3.4)	74 (2.9)	1262 (3.5)	>0.05
**Vigorous [N (%)]**								
More than once a week	10,133 (29.3)	1039 (24.0)	9094 (30.1)	<0.05	11,293 (29.1)	470 (18.6)	10,823 (29.8)	<0.05
Once a week	4983 (14.4)	492 (11.4)	4491 (14.9)	<0.05	5495 (14.1)	323 (12.8)	5172 (14.2)	<0.05
1–3 times a month	3989 (11.5)	443 (10.2)	3546 (11.7)	<0.05	4357 (11.2)	254 (10.0)	4103 (11.3)	>0.05
Hardly ever, or never	15,458 (44.7)	2360 (54.5)	13,098 (43.3)	<0.05	17,696 (45.6)	1485 (58.7)	16,211 (44.6)	<0.05
**Moderate [N (%)]**								
More than once a week	22,353 (64.7)	2618 (60.4)	19,735 (65.3)	<0.05	25,209 (64.9)	1309 (51.7)	23,900 (65.8)	<0.05
Once a week	4865 (14.1)	594 (13.7)	4271 (14.1)	>0.05	5422 (14.0)	357 (14.1)	5065 (14.0)	>0.05
1–3 times a month	2499 (7.2)	308 (7.1)	2191 (7.3)	>0.05	2733 (7.0)	222 (8.8)	2511 (6.9)	<0.05
Hardly ever, or never	4846 (14.0)	814 (18.8)	4032 (13.3)	<0.05	5477 (14.1)	644 (25.4)	4833 (13.3)	<0.05

Data from middle-aged and older population in Europe (2021) were used for analysis, participants were categorized into with/ without arthritis groups. All participants claimed have no OA/ RA in 2017.

Age and BMI are presented as mean ± SD·W9: the 9th data collection wave of the SHARE. *P*-value calculated from chi-square and logistic regression, *P*<0.05: a statistically significant level.

The cross-sectional analysis in Wave 9 included a total of 66,208 participants (Table S1), with an average age of 69.13 ± 12.23 years. Among these participants, 58.94 % reported to suffer from at least one metabolic disease 47.37 % (*n* = 31,361) reported HBP/HTN, 27.90 % (*n* = 18,475) reported HBC, and 15.17 % (*n* = 10,042) reported DM/HBC, 9.10 % reported RA and 18.50 % reported OA/other rheumatism. The prevalence of arthritis increased with the number of MDs (Table 3). Specifically. Among participants who reported engaging in PA, 56.20 % stated to engage in vigorous PA, while 86.05 % stated to engage in moderate PA.

**Table 3:** Associations between metabolic diseases and new-onset arthritis among middle-aged and older participants in Europe (2017–2021), with hazard ratio (HRs) and 95 % confidence intervals (95 % CI).

**Variables**	**OA/other rheumatism**			**RA**		
	**All**	**50–65**	**>65**	**All**	**50–65**	**>65**
**MDs (N)**						
MDs (0)	1.00	1.00	1.00	1.00	1.00	1.00
MDs (1)	1.090 (1.016–1.170)	1.120 (0.971–1.292)	1.247 (1.144–1.360)	1.148 (1.045–1.262)	1.355 (1.098–1.672)	1.184 (1.064–1.318)
MDs (2)	1.167 (1.072–1.271)	1.177 (0.960–1.443)	1.333 (1.212–1.466)	1.332 (1.195–1.484)	1.753 (1.374–2.237)	1.329 (1.178–1.498)
MDs (3)	1.192 (1.033–1.376)	1.318 (0.906–1.920)	1.440 (1.261–1.644)	1.316 (1.100–1.573)	2.318 (1.614–3.33)	1.321 (1.089–1.601)

Data from middle-aged and older population in Europe (2017 and 2021) were used for Cox regression analysis.

Data presented as HR (corresponding 95 % CI); HRs were derived from Cox regression model and adjusted for Age, BMI and sex.

### 3.2 Risk of new-onset arthritis

After adjusting for confounding factors (Table 3), the cox regression model revealed that compared to participants without MDs, those with MDs had a significantly higher risk of new-onset OA/other rheumatism (one MD: HR = 1.090; two MDs: HR = 1.167; three MDs: HR = 1.192, *P* < 0.05) and RA (one MD: HR = 1.148; two MDs: HR = 1.332; three MDs: HR = 1.316, *P* < 0.05). When stratifying by age, in participants aged 50–65 years there was no significant association between the total number of MDs and the risk of new-onset OA/other rheumatism compared to those without MDs. However, among participants aged over 65 years a significant dose-response relationship was found between the number of MDs and the risk of new-onset OA/other rheumatism (one MD: HR = 1.247; two MDs: HR = 1.333; three MDs: HR = 1.440, *P* < 0.05). Regarding RA, among participants aged 50–65 years with MDs, a significant dose-response relationship was observed between the number of MDs and the risk of new-onset RA (one MD: HR = 1.355; two MDs: HR = 1.753; three MDs: HR = 2.318, *P* < 0.05). Among those over 65, there was a significant increase in the risk of new-onset RA (one MD: HR = 1.148; two MDs: HR = 1.332; three MDs: HR = 1.316, *P* < 0.05) compared to participants without MD.

### 3.3 Associations between lifestyle factors and arthritis

The adjusted regression model revealed that the associations between lifestyle factors and arthritis varied depending on arthritis type (Table 4). For RA, both vigorous and moderate physical activity were negatively associated with RA (*P* < 0.05), but smoking and alcohol consumption were not significantly associated with RA (*P* > 0.05). For OA/other rheumatism, engaging in vigorous and moderate PA once a week and high alcohol consumption were associated with lower OA/other rheumatism prevalence (*P* < 0.05), while never engaging in vigorous PA, moderate alcohol consumption, and smoking were associated with higher OA/other rheumatism prevalence (*P* < 0.05).

**Table 4:** Associations between metabolic diseases, lifestyle factors and arthritis among middle-aged and older participants in Europe (2021), with odds ratios (ORs) and 95 % confidence intervals (95 % CI).

**Variables**	**OA/other rheumatism**			**RA**		
	**All**	**50–65**	**>65**	**All**	**50–65**	**>65**
**Vigorous PA**						
More than once a week	1.00	1.00	1.00	1.00	1.00	1.00
Once a week	0.917 (0.855–0.984)	0.912 (0.812–1.024)	0.920 (0.841–1.007)	1.184 (1.073–1.305)	1.079 (0.914–1.275)	1.247 (1.103–1.409)
1–3 times a month	0.981 (0.909–1.059)	0.955 (0.840–1.085)	0.996 (0.905–1.096)	1.195 (1.076–1.327)	1.129 (0.942–1.354)	1.234 (1.083–1.406)
Hardly ever, or never	1.242 (1.176–1.311)	1.236 (1.126–1.356)	1.252 (1.17–1.339)	1.328 (1.229–1.436)	1.295 (1.131–1.484)	1.355 (1.230–1.492)
**Moderate PA**						
More than once a week	1.00	1.00	1.00	1.00	1.00	1.00
Once a week	0.928 (0.872–0.988)	0.904 (0.808–1.012)	0.944 (0.876–1.017)	1.178 (1.086–1.278)	1.026 (0.876–1.202)	1.245 (1.131–1.37)
1–3 times a month	0.971 (0.895–1.053)	1.012 (0.872–1.173)	0.959 (0.870–1.058)	1.348 (1.217–1.493)	1.243 (1.019–1.518)	1.398 (1.24–1.576)
Hardly ever, or never	0.989 (0.930–1.052)	0.899 (0.78–1.035)	1.023 (0.955–1.096)	1.574 (1.457–1.699)	1.618 (1.362–1.923)	1.589 (1.457–1.733)
**Smoking#**	1.302 (1.248–1.359)	1.214 (1.127–1.307)	1.341 (1.273–1.413)	1.020 (0.962–1.081)	1.153 (1.038–1.281)	0.961 (0.895–1.031)
**MDs (N)**						
MDs (0)	1.00	1.00	1.00	1.00	1.00	1.00
MDs (1)	1.311 (1.248–1.377)	1.432 (1.316–1.558)	1.240 (1.168–1.317)	1.536 (1.433–1.645)	1.663 (1.473–1.878)	1.468 (1.35–1.596)
MDs (2)	1.396 (1.320–1.477)	1.510 (1.355–1.683)	1.328 (1.243–1.419)	1.793 (1.662–1.934)	1.756 (1.510–2.044)	1.760 (1.611–1.924)
MDs (3)	1.487 (1.367–1.618)	1.869 (1.564–2.233)	1.366 (1.241–1.504)	1.907 (1.713–2.124)	2.216 (1.752–2.804)	1.799 (1.592–2.032)
**Alcohol consumption**						
Low	1.00	1.00	1.00	1.00	1.00	1.00
Middle	1.095 (1.012–1.185)	1.185 (1.056–1.331)	1.022 (0.917–1.140)	0.915 (0.815–1.029)	0.876 (0.730–1.052)	0.947 (0.813–1.102)
High	0.804 (0.704–0.917)	0.838 (0.668–1.053)	0.786 (0.669–0.925)	1.072 (0.910–1.263)	1.147 (0.861–1.529)	1.040 (0.852–1.269)

Data from middle-aged and older population in Europe (2021) were used for logistic regression analysis.

Data are presented as Odds ratios and 95 % Confidence interval (CI); #: no smoking as reference.

Upon stratifying by age, hardly ever or never participating in vigorous PA was positively associated with the prevalence of OA/other rheumatism and RA (*P* < 0.001). In the age group of 50–65 years, compared to low alcohol consumption, moderate alcohol consumption was positively associated with the prevalence of OA/other rheumatism (*P* < 0.05). Furthermore, compared to non-smokers, smoking was associated with higher RA prevalence in people of 50–65 years old (*P* < 0.05). In the population aged over 65 years, compared to low alcohol consumption, high alcohol consumption was associated with lower OA/other rheumatism prevalence (*P* < 0.05).

Furthermore, compared to having no MDs, the presence of one or multiple MDs was positively associated with the prevalence of OA/other rheumatism and RA (*P* < 0.001). Higher age, higher BMI, and female sex were positively associated with the prevalence of different types of arthritis (*P* < 0.001).

## 4 Discussion

This study integrates longitudinal and cross-sectional analysis to establish metabolic disease burden as a dose-dependent predictor of arthritis risk in Europeans ≥50 years, while revealing inverse associations with regular moderate-to-vigorous physical activity. Differential risk profiles emerged across arthritis subtypes and age cohorts regarding smoking/ alcohol exposure.

Our study not only identified that middle-aged and older adults with a higher number of MDs are more susceptible to arthritis but also further evidenced the causal relationship between metabolic diseases and arthritis due to the prospective nature of the longitudinal study. Cross-sectional studies have already pinpointed MDs as a risk factor for arthritis. An overall arthritis prevalence of 31.4 % was found in participants aged ≥45, with hypertension being identified as a risk factor for arthritis (Li et al., 2015). Moreover, a prospective cohort study from the UK Biobank, following 370,311 participants over 12.48 years, demonstrated that individuals with MDs had a higher risk of developing OA (Zhang et al., 2024). Our research provides further support for the association between MDs and an elevated risk of arthritis incidence and prevalence in a European population. After stratifying by age, we discovered a dose-response effect between MDs and the risk of OA/other rheumatism in the >65 age group, and RA risk in the 50–65 age group. Metabolic diseases can promote the development of arthritis not only by damaging cartilage formation through excessive lipid deposition and increasing the accumulation of advanced glycation end-products in joint tissues (Aspden et al., 2001; Zhuo et al., 2012) but also elevate the risk to develop depression, cardiovascular diseases, and other comorbidities (Santos-Moreno et al., 2022; Feng et al., 2023). In this study, we found that a majority of participants reported at least one metabolic disease, underscoring the importance of early prevention of MDs and reducing the incidence of OA and RA. However, no association was found between MDs and the risk of OA/other rheumatism in the 50–65 age group, suggesting that the impact of MDs on OA/other rheumatism may vary by age group. This also indicates a need to focus on age, BMI, and female sex for the prevention of new-onset OA/other rheumatism.

Although physical activity has been recognized as an effective means of managing arthritis (Gwinnutt et al., 2022), the association between PA and arthritis varies across types of arthritis and age groups. It is worth noting that 99.8 % of the participants in the ninth wave of the SHARE project provided information about PA, compared with only 18.1 % of the subjects in the seventh wave, allowing for a more comprehensive assessment of the relationship between PA and arthritis. PA was positively associated with RA across different age groups, it was also observed in a meta-analysis evaluating the impact of cardiovascular and strength exercises on inflammation, joint damage, and symptoms in patients with inflammatory rheumatic diseases: PA was found to significantly improve the patients' disease activity scores, joint damage, and inflammation status (Silje Halvorsen et al., 2017). Recreational PA was observed to reduce the risk of occurrence of RA in a 21.4-year follow-up study of 113,366 American women aged 36.5 years at baseline (Liu et al., 2019). Our study supports that high PA levels were associated with lower risk of RA in the middle-aged and older population. The association between PA and OA is different from the effects of PA on RA, as PA may lead to mechanical loading of cartilaginous structures which in some cases can also promote the development of OA (Guilak, 2011). However, our data clearly show that individuals who hardly ever exercise are at higher risk to develop OA. This fits to the observation that the decline in muscle strength that comes with aging and reduced physical activity also has been identified as a risk factor for the development of OA (VillafaÑe et al., 2019). Furthermore, an intervention trial in American individuals aged 60 and above found that, compared to those engaging in less PA, patients engaging in more PA experienced reductions in inflammatory markers such as IL-6 and TNF-α, improving pain and function in OA patients (Runhaar et al., 2019). Thus, to improve the management of arthritis and joint function participation in PA should be encouraged.

Smoking was clearly associated with OA among all ages, while it was associated with RA in the 50–65 age group only, possibly indicating distinct pathogenic mechanisms between the two types of arthritis. Smoking can significantly accelerate telomere shortening and aging in cartilage cells due to oxidative stress, while also impairing oxygen transport capacity, reducing oxygen supply to joint cartilage, and thus promoting the development of OA (Liu et al., 2022; Wang et al., 2023). In RA, smoking may have a less pronounced but still detrimental effect. Smoking was found to be a predictor of severe extra-articular disease manifestations of RA in American individuals over 46-years-old (Turesson et al., 2003). Additionally, cigarette smoke condensate can induce the upregulation of pro-inflammatory cytokines (such as IL-1α, IL-6) in fibroblast-like synoviocytes (Shizu et al., 2008). In contrast to our results, a case-control study of Swedish adults aged over 50 observed a significant association between smoking and increased risk of new-onset RA (Kokkonen et al., 2017). This discrepancy underscores the need for targeted investigations to clarify the potential causal relationship between smoking and RA development in older populations. Similar to the effect of smoking the impact of alcohol may depend on arthritis type. While we observed no association between alcohol consumption and RA, for OA middle alcohol consumption increased and high alcohol consumption decreased the risk compared to low alcohol consumption. In addition to the amount, it may also be the type of the alcoholic beverage that is related to positive or negative effects on OA. A positive dose-response relationship between beer consumption and OA was established by a case-control study involving 3171 British participants aged 45 to 86, whereas wine consumption was negatively associated with knee osteoarthritis (Muthuri et al., 2015). However, the database of SHARE only recorded the frequency of alcoholic beverage intake over the past three months, making it impossible to conduct subgroup analyses to further explore the source of the negative correlation between high alcohol consumption and OA. A cross-sectional study of 188 newly diagnosed RA patients and 192 asymptomatic participants aged over 50 in Netherland found no association between alcohol and the severity of arthritis inflammation, which fits to our results and underlines that alcohol has no major impact on RA (Lukas et al., 2018). Although high alcohol consumption was correlated with lower risk of OA in our study, this does not imply that alcohol consumption should be recommended or is in general good for patients' health. On the contrary, smoking and alcohol consumption are major risk factors for the global disease burden and can harm health through multiple pathways including increasing blood pressure levels and the risk of stroke (Millwood et al., 2019; Collaborators GA, 2018).

Findings from both longitudinal and cross-sectional studies have revealed that higher BMI, higher age, and female sex correlate with the risk of arthritis. Obesity has been found to increase the risk of OA and RA occurrence after 8.66 years of follow-up among 20,859 American participants with an average age of 53.74 years. This risk elevation may be attributed to higher mechanical stress at the joints, leading to more rapid degenerative changes, while the inflammatory mediators associated with obesity further the progression of arthritis (Guilak, 2011; Nan et al., 2024). The higher age and female sex were identified as risk factors for the development of new-onset arthritis in a four-year longitudinal study involving 17,314 Chinese individuals aged 45 and over (Li et al., 2015). In our study, the average BMI of participants already classified as overweight (World Health O, 2023), which highlights the importance of weight management for promotion of joint health in the European middle-aged and older populations, particularly in females.

We acknowledge our study with limitations. Firstly, SHARE used questionnaires to collect self-reported information from participants, which means that potential reporting biases can occur. Secondly, due to the setting of the questionnaire, the question combines OA with other forms of disease into the option “OA/other rheumatism”. Hence, the statistics regarding prevalence and incidence of OA/other rheumatism require cautious interpretation. Excessive physical activity may contribute to the progression of lower limb osteoarthritis. However, current analytical limitations arise from the concurrent presence of OA in multiple anatomical sites, as existing databases lack detailed classification of OA locations. This absence of site-specific OA data could potentially confound the results of the analysis. Finally, our research includes individuals aged 50 and above; therefore, the findings cannot be generalized to younger populations.

## 5 Conclusion

Our findings demonstrate that metabolic diseases elevate risks of OA/ other rheumatism and RA in European adults aged ≥50. The risk of OA/ other rheumatism increases with smoking and hardly ever or never exercise. Similarly, the risk of RA is significantly reduced by higher levels of physical activity. Additionally, this study highlights that female sex demonstrates a more pronounced association with arthritis prevalence compared to high BMI. These findings advocate implementing metabolic-integrated prevention strategies, smoking-exercise focused public health interventions, and sex-specific screening protocols.

## Availability of data and materials

The data that support the findings of this study are available from SHARE Wave 7 (https://doi.org/10.6103/SHARE.w7.800) and Wave 9 (https://doi.org/10.6103/SHARE.w9.900) but restrictions apply to the availability of these data, which were used under license for the current study, and so are not publicly available. Data are however available from the authors upon reasonable request and with permission of SHARE project.

**Clinical trial registration**: not applicable.

## CRediT authorship contribution statement

**Fanji Qiu:** Writing – original draft, Methodology, Formal analysis, Data curation, Conceptualization. **Jinfeng Li:** Writing – review & editing, Methodology. **Liaoyan Gan:** Writing – review & editing, Methodology. **Kirsten Legerlotz:** Writing – review & editing, Supervision.

## Consent for publication

Not applicable.

## Author statement

We confirm that each individual named as an author meets the journal's criteria for authorship. F.Q. conceived and designed the study, supervised by K.L.; J.L., L.G. and F.Q. conducted the statistical analysis and interpretation of the data; F.Q. drafted and K.L. reviewed the manuscript. All authors have read and approved the final version.

## Ethical approval and consent to participate

SHARE has been repeatedly reviewed and approved by the Ethics Committee of the University of Mannheim. Written informed consent was obtained from all participants.

## Funding

Not applicable.

## Declaration of competing interest

The authors declare that they have no known competing financial interests or personal relationships that could have appeared to influence the work reported in this paper.

## Data Availability

Data will be made available on request.

## Appendix A Supplementary data

The supplementary document presents the screening process of the participants, the demographic information of the 2021 SHARE project participants, and the specific questions in the questionnaire for information collection. Image 1
