# Supplementary material for: Metabolic diseases and lifestyle factors affect arthritis incidence in old Europeans - a cross analysis from the SHARE project
Source: Prev Med Rep. 2025 Apr 28;54:103089. doi: 10.1016/j.pmedr.2025.103089 (PMC12084502; doi:10.1016/j.pmedr.2025.103089)
Supplement: Supplementary file 1 — The supplementary document presents the screening process of the participants, the demographic information of the 2021 SHARE project participants, and the specific questions in the questionnaire for information collection. [file mmc1.docx]

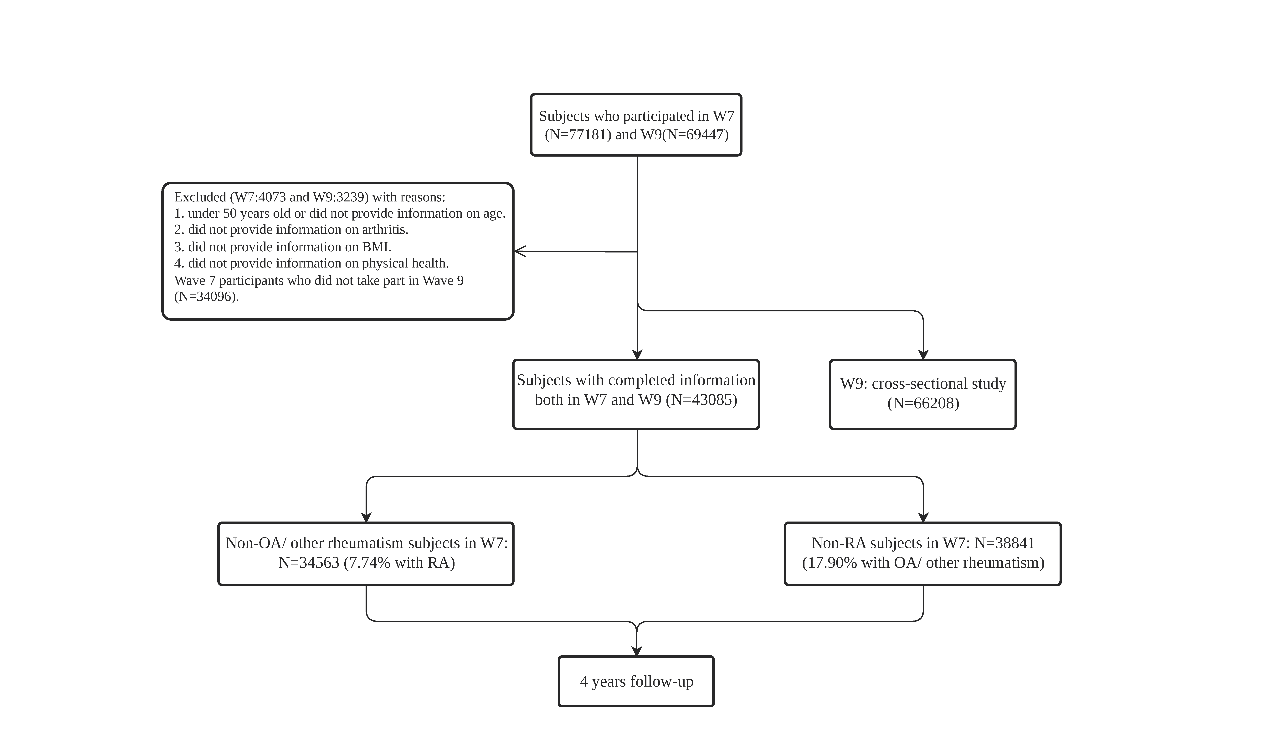


Figure S1. Flow diagram of the study population

Table S1. The Characteristics of patients by number of MDs (W9)

| **Variables** | **No. of MDs** | | | | |
| --- | --- | --- | --- | --- | --- |
|  | **0** | **1** | **2** | **3** | **All** |
| **N (%)** | 27187 (41.06) | 22070 (33.33) | 13045 (19.70) | 3906 (5.90) | 66208 |
| **Age [y]** | 66.79 ± 9.65 | 70.06 ± 16.17 | 71.49 ± 8.89 | 72.37 ± 8.59 | 69.13 ± 12.23 |
| **Female [N (%)]** | 15484 (57.0) | 12714 (57.6) | 7335 (56.2) | 2108 (54.0) | 37641 (56.9) |
| **MDs [%]** |  |  |  |  |  |
| HBP/HTN | 0 | 15280 (69.2) | 12175 (93.3) | 100 | 31361 (47.4)) |
| HBC | 0 | 4740 (21.5) | 9829 (75.3) | 100 | 18475 (27.9) |
| DM/ HBS | 0 | 2050 (9.3) | 4086 (31.3) | 100 | 10042 (15.2) |
| **Arthritis [N (%)]** |  |  |  |  |  |
| OA/other rheumatism | 3879 (14.3)^a^ | 4465 (20.2)^b^ | 2956 (22.7)^c^ | 978 (25.0)^d^ | 12278 (18.5) |
| RA | 1591 (5.9)^a^ | 2226 (10.1)^b^ | 1655 (12.7)^c^ | 572 (14.6)^d^ | 6044 (9.1) |
| **BMI [kg/m²]** | 26.02 ± 4.29 | 27.48 ± 4.68 | 28.47 ± 4.87 | 29.67 ± 5.40 | 27.20 ± 4.75 |
| **Smoking [%]** | 11109 (40.9) | 8813 (39.9) | 5303 (40.7) | 1607 (41.1) | 26832 (40.5) |
| **Alcohol consumption [N (%)]** |  |  |  |  |  |
| Low | 963 (3.5) | 688 (3.1) | 427 (3.3) | 84 (2.2) | 2162 (3.3) |
| Middle | 2555 (9.4) | 1759 (8.0) | 850 (6.5) | 239 (6.1) | 5403 (8.2) |
| High | 23669 (87.1) | 19623 (88.9) | 11768 (90.2) | 3583 (91.7) | 58643 (88.6) |
| **Vigorous [N (%)]** |  |  |  |  |  |
| More than once a week | 10365 (38.1) | 6665 (30.2) | 3087 (23.7) | 678 (17.4) | 20786 (31.4) |
| Once a week | 3890 (14.3) | 3161 (14.3) | 1697 (13.0) | 428 (11.0) | 9176 (13.9) |
| 1–3 times a month | 2925 (10.8) | 2465 (11.2) | 1455 (11.2) | 402 (10.3) | 7247 (11.0) |
| Hardly ever, or never | 10016 (36.8) | 9779 (44.3) | 6806 (52.2) | 2398 (61.4) | 28999 (43.8) |
| **Moderate [N (%)]** |  |  |  |  |  |
| More than once a week | 18937 (69.7) | 14549 (65.9) | 7885 (60.4) | 2014 (51.6) | 43385 (65.5) |
| Once a week | 3560 (13.1) | 3042 (13.8) | 1803 (13.8) | 581 (14.9) | 8986 (13.6) |
| 1–3 times a month | 1713 (6.3) | 1558 (7.1) | 989 (7.6) | 342 (8.8) | 4602 (7.0) |
| Hardly ever, or never | 2977 (11.0) | 2921 (13.2) | 2368 (18.2) | 969 (24.8) | 9235 (13.9) |

a, b, c, d: significant differences in prevalence, with significant differences between different letters and no significant differences for the same letter.

Questionnaire to collect information

The collection of participant information was sourced from questionnaires. Participants were asked: “Has a doctor ever told you that you had/ Do you currently have OA/ other rheumatism, Rheumatoid arthritis; diabetes mellitus or high blood sugar; high blood cholesterol; high blood pressure or hypertension? [With this we mean that a doctor has told you that you have this condition, and that you are either currently being treated for or bothered by this condition.]. Information on smoking was collected with the question: “Ever smoked daily?”. Each of the aforementioned questions included two response options: 0 (Not selected, indicating the absence of the condition) or 1 (Selected, indicating the presence of the condition). Information regarding participants' alcohol consumption was collected through the questionnaire item: "How often did you have 6 or more drinks on one occasion in the last 3 months?" Based on the participants' responses, alcohol consumption was categorized into three levels: high (daily or almost daily, 5 or 6 days in one week, and 3 or 4 days in one week); middle (once and twice in one week, once or twice in one week), and low (less than once in one month, not at all in the last 3 months). Information on PA was collected with the question: “How often do you engage in vigorous physical activity (sports, heavy housework, or a job that involves physical labor)/ moderate physical activity (gardening, cleaning the car, or doing a walk?)”. Participants' frequency of PA was classified into four categories: more than once a week, once a week, one to three times a month, and hardly ever or never. Due to the setting of the questionnaire, some physical health conditions were listed in combination and then counted together, including “OA and other rheumatism”, “high blood pressure or hypertension”; “diabetes or high blood sugar”.
